# Supplementary material for: Are carbon emissions trading and green financial instruments synergistic? -Comprehensive quantitative research based on content analysis
Source: PLoS One. 2024 Mar 7;19(3):e0298601. doi: 10.1371/journal.pone.0298601 (PMC10919601; doi:10.1371/journal.pone.0298601)
Supplement: S1 Data — (ZIP) [file pone.0298601.s001.zip › Data/地级市Z_F工作报告.zip!/╔╜╢1⁄2/╢1⁄2╙¬╩╨/└┤╫╘╣1⁄2╓┌║┼í╛┬φ┐╦╩2╛▌═°í┐_2012.docx]

各位代表：

现在，我代表市人民政府向大会报告政府工作，请予审议，并请市政协委员和其他列席会议的同志提出意见。

一、本届政府工作回顾

市第六届人民政府就职四年多来，在省委、省政府和市委的正确领导下，全市上下深入贯彻落实科学发展观，抢抓机遇、积极作为，改革创新、攻坚克难，大力推进黄蓝经济区建设，“十一五”规划任务圆满完成，“十二五”规划顺利实施，全市经济社会发展取得显著成绩。

(一)积极应对困难挑战，经济保持平稳较快发展。2008年国际金融危机爆发以来，国内外经济形势复杂多变，对我市经济发展产生严重影响。我们认真贯彻国家宏观调控政策，采取综合措施，沉着应对，保持了经济平稳较快发展。2011年全市生产总值达到2676.4亿元，年均增长13.1%。工业效益和竞争力大幅提高，规模以上工业产值、主营业务收入双双突破8000亿元，年均分别增长20.9%、26.6%；主营业务收入过10亿元企业达到119家，过百亿元企业达到16家；实现工业利税1653.2亿元、利润999.7亿元，年均分别增长19.3%、15.6%。实现地方财政收入135.27亿元，年均增长22.5%。投资、消费不断扩大，全社会固定资产投资达到1574.4亿元，年均增长26%；社会消费品零售总额达到448.9亿元，年均增长19.6%。金融机构各项存款余额1927.7亿元、贷款余额1439.6亿元，年均分别增长22.7%、23.3%。

(二)全力实施两大国家战略，黄蓝经济区建设拉开新格局。主体产业区建设快速突破。东营经济技术开发区，引进建设广汽吉奥汽车等过亿元项目116个，汽车及零部件、电子信息、新能源、有色金属等6个百亿级产业集群已具雏形，产值突破千亿元，升级为国家级开发区。东营港经济开发区，基础设施配套加快推进，五大生态工业园和三大物流园建设进展顺利，被列为中国石油化工产业区。黄河口生态旅游区，规划建设游客综合服务中心、湿地公园等重点项目，成为国家4A级景区。生态高效农业区，现代渔业示范区建成养殖区20万亩，综合配套服务区和滨海蓝镇建设顺利推进；高效生态农业示范区被列为省级农业高新技术产业示范区，现代畜牧业示范区升级为省级示范区。生态环境建设力度加大。“三网”绿化工程完成绿化面积87万亩，启动生态林场建设试点，成为国家现代林业建设示范市。实施了湿地修复工程，黄河刁口河流路恢复过水。强力推进节能减排，全面完成省政府下达的目标任务。建成12个重点循环经济项目。加强水气污染治理，新建改造污水处理厂6座，中心城区15家工业企业基本完成搬迁或关停任务。基础设施建设快速推进。东营港一期扩建工程建成通航并实现外轮开放，八大业主码头和港池改扩建工程加快实施。胜利机场4D级改扩建工程全面竣工，新航站楼投入使用。荣乌高速公路东营段建成通车，德大铁路东营段和新博公路建设加快推进。东营港防潮堤开工建设，滨海生态城防潮堤前期工作进展顺利。电力、通信等设施进一步完善。

(三)加大经济结构调整力度，产业层次大幅提升。积极推进区域经济结构调整，石油工业稳步发展，地方经济快速壮大，油地结构调整为3：7；市属经济加快发展，县域经济实力不断增强。大力推进产业结构调整，三次产业结构由2007年的3.7：78.1：18.2调整为3.7：71.6：24.7。加快发展环境友好型工业，传统优势产业改造提升成效显著，先进制造业和高新技术产业快速突破，战略性新兴产业和海洋产业开始起步，新口径高新技术产业比重达到30.7%。积极发展现代服务业，文化旅游、现代物流、服务外包等产业快速发展，创建成为中国优秀旅游城市，服务业增加值年均增长14.1%。加大自主创新力度，规划建设黄河三角洲可持续发展研究院、国家大学科技园等创新平台，连续保持全国科技进步先进市称号，被评为全国知识产权工作示范城市。大力实施质量兴市战略，新增中国驰名商标12件、山东名牌26个。加强人才队伍建设，成立了中德农牧业技术培训中心，引进培养了一批产业领军人才和专业技能人才。

(四)扎实推进“三农”工作，新农村建设取得新成效。全面落实强农惠农富农政策，市级财政“三农”投入累计达到33.9亿元，年均增长10.6%。粮棉菜生产连年丰收。渔业、畜牧业等优势产业加快发展，特色水产品养殖达到185万亩，标准化畜牧养殖小区达到854个。休闲观光农业、工厂化食用菌等新兴产业快速发展。积极推进农业产业化，龙头企业达到530家，农民专业合作社发展到620家。农业机械化水平显著提高。东营被列为国家现代农业示范区。着力提高农民科技素质，累计培训农民40万人次。加强农业农村基础设施建设，改造中低产田37.2万亩、灌渠12条，治理骨干河道258公里，新建改造农村公路1939公里，新建农房6万户，改造危房1.5万户，解决了39.5万农村人口的饮水安全问题，建成新型农村社区150个，农村生产生活条件明显改善。

(五)加快宜居城市建设，城镇面貌发生巨大变化。中心城新一轮总体规划修编完成，对广利河进行了综合治理，黄河国际会展中心、清风湖公园等项目建成投用，奥体中心、大剧院等项目建设进展顺利。东城旧区改造开始启动。西城改造步伐加快，“六路一河一桥五公园”及15处街心绿地建设基本完工。城市道路交通、供水、供暖、供气等市政公用设施不断完善。大力推进城市绿化、美化、亮化，新增绿地696万平方米，城市绿化覆盖率达到39.7%，创建成为国家园林城市。加快推进新型城镇化，促进中心城、次中心城、卫星城镇协调发展，城镇化水平提高到60.97%。

(六)不断深化改革开放，发展动力活力明显增强。加大金融创新力度，引进8家股份制银行，组建3家村镇银行和3家农村商业银行，市商业银行更名为东营银行，城市基础设施建设债券发行融资12亿元。积极推进未利用地开发管理改革试点，在全省率先启动未利用地开发项目。大力支持企业改革，民营经济活力不断增强，5家企业成功上市。深化农村综合改革，土地流转制度、国有农场和集体林权制度改革取得明显成效。加快医药卫生体制改革，圆满完成三年医改任务，基本药物制度全面实施，公立医院改革走在全省前列，市人民医院与省立医院合作全面展开。扎实推进文化体制改革，组建了市广播电视台、市演艺公司和文化市场综合执法机构。市县乡政府机构改革任务全面完成，事业单位分类改革稳步推进。加快发展开放型经济，进出口总值达到102.2亿美元，其中出口43.6亿美元，比2007年分别增长2.9倍和2.1倍；引进世界500强企业10家，累计利用外资6.95亿美元。加快“走出去”步伐，境外投资、对外工程承包和劳务合作快速增长。

(七)着力保障改善民生，社会保持和谐稳定。千方百计增加城乡居民收入，城镇居民人均可支配收入达到27343元，年均增长10.1%；农民人均纯收入达到10025元，年均增长14.3%。实施积极的就业政策，累计新增城镇就业16.4万人次，农村劳动力转移就业22.4万人次，城镇登记失业率控制在2%以内。努力提高社会保障水平，企业最低工资标准提高到每月1240元，各级财政对城镇居民医保、新农合补助标准提高到210元以上，城市低保标准提高到每月380元以上，农村低保、五保集中供养标准分别提高到每年2060元、4200元，城乡居民养老保险实现全覆盖。建设保障性住房5.1万套、棚户区安置房7548套。支持油田新建职工住房4万套，补办房产证5000户。每年集中实施十件民生实事，改善了群众生产生活条件。坚持教育优先发展，基本实现城乡免费义务教育，实施了中小学校舍安全改造等重点工程，新建改造幼儿园231所，市技师学院新校区和胜利一中高中部迁建一期工程竣工投用。大力发展卫生事业，改造县医院、乡镇卫生院23处，建成村卫生室和城市社区卫生服务机构725处。繁荣发展文化事业，规划建设了一批文化设施，成功举办了黄河口文化艺术节等节庆活动，群众文化生活日益丰富。加强精神文明和民主法制建设，文明创建活动深入开展，普法和依法治理扎实推进。广泛开展全民健身运动，成功举办了四届黄河口国际马拉松赛。人口计生工作成效显著，低生育水平持续稳定。国防教育和双拥共建深入开展，连续三次荣获全国双拥模范城称号。创建成为全国残疾人工作示范城市。大力推进平安东营建设，加强和改进信访工作，强化社会治安综合治理，加大打黑除恶力度，有力地维护了社会稳定。高度重视安全生产，加强食品药品安全监管，健全完善应急管理体系，保障人民群众健康安全。对口支援工作取得明显成效。新闻出版、外事侨务、人民防空、防震减灾、民族宗教、社会科学、妇女儿童、老龄、青少年、慈善和红十字会等事业健康发展，对台、统计、档案、史志、仲裁、气象、无线电管理等工作都取得新成绩。

(八)加强政府自身建设，行政效能不断提高。认真落实依法行政实施纲要，加快转变政府职能，依法行政意识不断增强，行政行为进一步规范。深化行政审批制度改革，精简审批项目，推行网上审批，政府工作效率和服务水平不断提高。注重畅通社情民意渠道，开通了市长公开电话。深入贯彻实施公务员法，公务员队伍整体素质明显提升。坚持从严治政，全面落实党风廉政建设责任制，强化行政监察和审计监督，严肃查处违纪违法案件，反腐倡廉建设得到进一步加强。

各位代表，四年多来，经过全市人民的共同努力，东营经济综合实力大幅提升，质量效益不断提高，社会事业全面进步，城乡面貌深刻变化，人民生活明显改善。今天的东营，已经站在了一个基础坚实、蓄势待发的新起点上。这样的大好局面，是省委、省政府和市委正确领导、科学决策的结果，是市人大、市政协和社会各界有效监督、大力支持的结果，是全市上下、油地军校同心同德、团结奋斗的结果。在此，我代表市人民政府，向辛勤工作在各条战线上的广大干部群众，向各民主党派、工商联、各人民团体和各界人士，向驻东营人民解放军、武警官兵和中央、省驻东营各单位，向所有关心、支持东营发展的海内外朋友，致以崇高的敬意，表示衷心的感谢！

在肯定成绩的同时，我们也清醒地看到，我市经济社会发展还面临不少矛盾和问题，主要是：经济结构性矛盾仍然突出，传统产业比重过大，服务业比重偏低，自主创新能力不强，转方式调结构任务艰巨；生态建设和环境污染治理的任务十分繁重，节能减排压力巨大；对外开放程度不高，利用外资规模小；城乡之间、区域之间发展不够平衡，基础设施和城市功能还不完善；社会事业发展滞后，居民收入有待进一步提高，一些群众关心的民生问题还没有得到有效解决；各类社会矛盾纠纷仍然较多，社会管理创新水平亟待提高。政府自身建设还需要进一步加强，主要是思想不够解放，创新意识不强，工作效率不高，工作作风和服务质量有待进一步改进。对上述问题，我们一定要高度重视，采取有效措施，切实加以解决。

二、今后五年的目标任务

今后五年，是东营加快实施黄蓝国家战略、全面建设小康社会的关键时期，是深化改革开放、加快转变经济发展方式的攻坚时期。综观国内外形势，机遇与挑战并存，机遇大于挑战，我们仍处于大有可为的重要战略机遇期。经过二十九年特别是近几年的发展，我市综合实力大幅提升，内生动力明显增强，发展基础更加坚实；黄蓝国家战略在这里融合交汇，一体推进，为我市创造了更为广阔的发展空间；尤为重要的是，前不久召开的市第五次党代会，明确提出了今后五年的总体思路和奋斗目标，掀开了建设生态文明典范城市的新篇章，为东营的发展注入了新的强大动力。同时也要看到，国内外形势复杂多变，影响发展的不稳定不确定因素仍然较多，区域间竞争压力加大。我们必须准确把握东营经济社会发展的阶段性特征，深刻认识面临的历史机遇和风险挑战，切实增强机遇意识、忧患意识、责任意识，充分利用各种有利条件，积极克服各种不利因素，努力把各项工作做得更好。

根据市第五次党代会作出的部署，今后五年政府工作的总体要求是：高举中国特色社会主义伟大旗帜，以邓小平理论和“三个代表”重要思想为指导，深入贯彻落实科学发展观，紧紧围绕主题主线，全力实施黄蓝国家战略，着力调整优化结构，着力加强生态建设，着力深化改革开放，着力保障改善民生，促进经济长期平稳较快发展和社会和谐稳定，努力建设经济繁荣发达、城市秀美宜居、社会文明和谐、人民富裕幸福的生态文明典范城市。

今后五年经济社会发展的主要目标是：在优化结构、提高效益、降低消耗、保护环境的基础上，生产总值年均增长12%以上，全社会固定资产投资年均增长18%，社会消费品零售总额年均增长17%，进出口总值年均增长12%，地方财政收入年均增长14%以上，城镇居民人均可支配收入年均增长11%以上，农民人均纯收入年均增长12%以上。

经过五年的奋斗，实现上述目标，东营的发展将跃上一个大的台阶。到那时，经济综合实力大幅提升，高端生产要素加快聚集，发展方式明显转变，生态产业形成规模，成为全省重要的经济增长极。到那时，城市功能更加完善，城乡一体化进程加快，人居环境明显改善，森林环抱、湿地相间、水系环绕、绿茵棋布，成为国家生态园林城市。到那时，社会文明程度明显提高，人民群众权益和社会公平正义得到保障，人与人之间相互尊重、融洽相处，社会更加和谐稳定，成为全国文明城市。到那时，社会就业更加充分，社会保障水平明显提高，家庭财富显著增加，人民生活更加富裕，幸福感自豪感普遍增强。那时的东营，必将以现代化生态文明城市的神奇魅力展现在世人面前，令人留恋，令人向往！

各位代表，做好今后五年的工作，最根本的是加快建设生态文明典范城市。这是实施黄蓝国家战略的内在要求，是推动城市转型发展的必然趋势，是全市人民的共同期盼。我们要坚定不移地走生态文明发展道路，牢记使命，不负重托，积极作为，扎实苦干，奋力开创生态文明典范城市建设新局面，推动东营科学发展、率先发展、跨越崛起。工作中，要突出把握好六个方面：

——突出实施国家战略，着力建设黄蓝经济区。实施国家战略建设黄蓝经济区是全市第一位的任务。我们务必抓住机遇，集中优势资源，凝聚各方力量，全力向前推进，使之成为建设生态文明典范城市的强大引擎。要加快突破市属经济，重点打造三个增长极。中心城区，优化空间布局和产业布局，提升城市服务功能，吸引聚集先进要素，打造现代服务业聚集区；东营经济技术开发区，集中培育一批具有核心竞争力的高端产业，奋力抢占未来发展制高点，打造全省乃至环渤海地区高新技术产业的聚集区；东营港经济开发区，按照港区城一体发展的思路，大力发展临港经济，打造重要的现代生态化工基地和国际物流港。要加快突破县域经济，支持各县区积极对接黄蓝国家战略，发挥优势，竞相发展，形成各具特色的经济隆起带；加大促强扶弱力度，支持强县强镇加快发展，以更有效的措施实施突破利津战略。要加快突破民营经济，强化政策扶持，倡导全民创业，培育一批科技型企业和小微企业；帮助骨干企业搞好战略策划，做大做强主业，打造一批大型民营企业集团。要坚定不移深化改革，全方位扩大开放，推进体制机制创新，提高开放型经济水平，为黄蓝经济区建设注入强大动力活力。

——突出经济转型，着力优化产业结构。深入实施高端高质高效产业发展战略，全面提升准入门槛，推动产业转型升级，加快构建具有高效生态特色的现代产业体系。加快工业结构优化升级，改造提升现有优势产业，着力发展先进制造业和战略性新兴产业，高新技术产业比重达到36%以上。稳步发展石油工业，支持胜利油田增储上产。推动服务业跨越发展，着力培育特色集聚区、骨干企业和名牌产品，促进服务业扩大总量、优化结构、提升层次，服务业比重每年提高2个百分点以上。积极发展现代农业，突出发展生态渔业、生态畜牧业、绿色高端种植业和休闲观光农业，加快推进农业区域化布局、标准化生产和产业化经营，促进农业增效、农民增收。坚持海陆统筹、有序开发、一体发展，提高海洋开发管理能力，大力发展海洋经济，构建现代海洋产业体系。深入实施科教兴市、人才强市战略，提高自主创新能力，建设创新型人才队伍，推动经济发展向创新驱动转变，建成国家创新型城市。

——突出生态建设，着力构筑生态系统。建设生态文明典范城市首要的是加强生态建设。要挖掘和整合自然生态资源，提升生态环境质量，以增绿、活水、治污、减排为重点，加快构筑特色鲜明的生态系统，彰显东营生态之美。增绿，就是大搞植树造林，加快生态林场建设，搞好“三网”绿化，建设林海绿洲。活水，就是引黄河水入城，贯通水网水系，搞好水库、湖泊、湿地开发利用，打造生态循环水系。治污，就是深入推进水气污染治理，加强源头控制，完善环保设施，加快重点流域、重点区域整治，让东营的天更蓝、水更清、空气更新鲜。减排，就是强力推进节能减排，加快企业技术改造，淘汰落后产能，大力发展循环经济，全面建设生态社会，努力实现绿色低碳发展。

——突出新型城镇化，着力推进城乡协调发展。按照黄河三角洲中心城市的定位，高起点大手笔规划，高标准高水平建设，实施生态对接，促进组团发展，努力打造秀美宜居、富有魅力的生态之城。改造提升中心城，坚持旧区改造与新区突破并举，全面推进深度开发，加快东城环境提升和西城改造，完善城市功能，打造城市精品，提升城市品位。规划建设滨海生态城，按照低碳生态、城水相融、城港区联动的原则，科学规划，有序建设，打造集观光旅游、休闲度假、滨海居住为一体的国际化现代化新城区；以中日生态城建设为突破口，带动新城开发。加快建设东港新城，完善生活居住、旅游休闲、综合服务等功能，建成东营港及临港产业区的服务中心。积极推进县城、小城镇和新型农村社区建设，加快基础设施向农村延伸、公共服务向农村覆盖，以城带乡、以工促农，形成城乡一体化发展新格局。

——突出发展环境建设，着力增强区域竞争力。环境是发展的生命线。必须牢固树立环境也是生产力的理念，大力优化发展环境，加速优质生产要素聚集，打造最佳“投资洼地”。着力优化发展“硬环境”，加强交通、水利、能源、信息等重大基础设施建设，重点构建立体交通网络，实现东营与胶东半岛、省会城市群经济圈、环渤海城市的紧密连接，增强发展支撑能力。加强服务型政府建设，进一步完善促进经济社会发展的政策体系，积极向上争取支持，努力打造一流的政策环境；切实减轻企业负担，落实结构性减税措施，清理和规范涉企收费，认真执行重大项目直通车、服务承诺、限时办结等制度，努力打造一流的政务环境；全面规范行政执法，依法规范市场经营秩序，引导各类市场主体依法经营、公平竞争、诚实守信，努力打造一流的法治环境；严厉打击各种霸头和黑恶势力，加大对建筑市场等重点行业的治理力度，努力打造一流的社会环境。

——突出以人为本，着力保障改善民生。把保障和改善民生作为根本出发点和落脚点，不断加大民生投入，提高人民群众的幸福感和满意度。实施更加积极的就业政策，统筹做好城镇新增劳动力就业、农村富余劳动力转移就业、下岗失业人员再就业工作。加大收入分配调节力度，努力做到居民收入增长与经济发展同步，劳动报酬增长与劳动生产率提高同步。加强和改进公共服务，加快构建覆盖全体居民的终身教育体系、社会保障体系、医疗保障体系、住房保障体系和公共文化服务体系，努力满足人民群众的基本需求。加强和创新社会管理，做好新形势下的群众工作，建立科学有效的利益协调、诉求表达、矛盾调处和权益保障机制，扎实开展平安东营建设，创建国家安全发展示范城市，促进社会和谐稳定。油地军校团结发展是实施黄蓝国家战略、建设生态文明典范城市的优势所在、力量所在、希望所在。我们要高举团结协作共同发展的旗帜，不断完善合作机制，充分发挥各方优势，加快资源整合，携手推进生态建设、城市建设、重大基础设施建设和产业项目建设，共同开创区域繁荣发展新局面，共同建设和谐幸福新家园，共同谱写生态文明典范城市建设新篇章！

各位代表，未来五年是东营发展史上至关重要的时期。我们肩负的任务光荣而艰巨，前进的道路上还会遇到许多困难和挑战。只要我们全市上下、油地军校、社会各界凝心聚力，开拓奋进，就一定能够战胜一切艰难险阻、实现我们的奋斗目标，就一定能够在科学发展的道路上迈出更大步伐，就一定能够创造出无愧于时代、无愧于人民的光辉业绩！

三、2012年工作重点

今年是全面建设生态文明典范城市的开局之年，也是黄蓝经济区建设求突破、出亮点的关键一年。做好今年的工作，意义十分重大。我们要科学判断形势，准确把握大局，奋力抢抓机遇，乘势开拓前进，努力夺取经济社会发展的新胜利，以优异成绩迎接党的十八大胜利召开。

今年全市经济社会发展的预期目标是：生产总值增长12%，全社会固定资产投资增长20%，社会消费品零售总额增长17%，进出口总值增长10%，吸收外商直接投资较快增长，地方财政收入增长16%，城镇居民人均可支配收入增长11%，农民人均纯收入增长12%，城镇登记失业率控制在2.4%以内，居民消费价格涨幅控制在4%左右，人口自然增长率控制在4.6‰以内，完成省政府下达的节能减排任务。围绕实现上述目标，重点抓好八个方面的工作：

(一)把握宏观经济形势，在保持经济平稳较快发展上取得新成效。当前，国内外经济形势依然复杂严峻，发达经济体增长动力不足，世界经济复苏进程艰难曲折；国内经济发展不平衡、不协调、不可持续的问题还很突出，经济增长下行风险加大。我们必须积极应对环境变化，认真落实和用好国家宏观经济政策，努力保持经济平稳较快发展。

第一，抓投资促发展。充分发挥投资对扩大内需的重要作用，突出投资重点，提高投资效益，努力实现投资稳定增长。坚定不移地实施项目带动战略，重点抓好30个重大建设项目和364个投资亿元以上的重大产业项目，以项目带投入、促发展。着力调整优化投资结构，加大对高端高质高效产业、蓝色产业的投资力度，加大对社会民生、农业农村、生态环保、城市和重大基础设施建设的投资力度，严格控制“两高”和产能过剩行业投资扩张。加大招商引资力度，变招商引资为招商选资，重点加强与国内外大企业集团的战略合作，上接央企、外接世界500强，加快引进一批产业拉动力强、带动就业多、财税贡献大的项目，吸引一批跨国公司地区总部和功能性机构落户我市，提高利用外资的规模和质量。围绕黄蓝经济区规划、国家产业政策和省鼓励发展的重点产业，搞好项目论证，储备一批大项目。放宽市场准入，促进民间投资稳定增长。

第二，扩消费促发展。大力实施市民增收工程，着力增加城乡居民特别是低收入群众收入，提高居民消费能力。认真落实鼓励消费政策，积极培育文化、旅游、健身、养老、家政等消费热点，努力扩大农村消费规模。加强流通设施建设，深入实施“万村千乡”市场工程，加大农贸市场建设改造力度，改善居民消费条件。着力优化消费环境，严厉打击制售假冒伪劣商品行为，维护消费者权益。高度重视物价问题，实行社会保障救助标准与物价上涨挂钩联动，加强市场物价监管，确保市场供应充足、价格稳定。

第三，拓外需促发展。实施市场多元化战略，大力开拓国际市场，推动出口逆势增长。进一步优化出口产品结构，支持橡胶轮胎、石油装备等主导产品提高出口份额，建设电子信息、服务外包、新能源等新兴产业出口基地，培植新的出口增长点。深入实施科技兴贸战略，扩大高附加值产品出口，推动加工贸易转型升级。认真落实出口退税、出口信保、跨境贸易人民币结算等政策，积极应对国际贸易摩擦，为对外贸易营造便利条件。搭建对外经贸平台，办好国际石油石化装备展等大型展会。扩大先进技术装备、重要能源资源和节能环保产品进口。加快“走出去”步伐，鼓励有条件的企业到境外承包工程、投资办厂，拓展发展空间。

第四，保企业促发展。当前企业运行面临许多困难，必须强化扶持措施，主动搞好服务，保障企业健康发展。坚持有扶有控，进一步完善政银企合作机制，引导金融机构加大对符合产业政策、有市场需求的企业特别是中小微企业的信贷支持力度。加快推进区域集优债务融资试点，支持企业上市等直接融资。深入开展“品牌建设年”活动，抓好“名家、名企、名品”宣传，扩大市场占有率。引导企业加强管理创新，提高科学管理水平。认真落实促进民营经济加快发展的政策措施，支持民营企业转型升级，不断提高发展质量和效益。高度重视企业家队伍建设，加大培养培训力度，建立完善长期激励机制，为他们学习成长、发展事业提供良好条件。

第五，强财税促发展。加大高效财源建设力度，完善税源管控体系，依法加强税收和非税收入征管，保持财政收入稳定增长。优化财政支出结构，实施精细化管理，尽可能把有限的财政资金向“三农”倾斜、向社会保障倾斜、向社会事业倾斜，严格控制一般性支出，集中财力办大事、保重点。加强财税政策研究，加大争取和挖潜力度，拓展财税增长空间。继续深化部门预算、国有资产管理、政府采购、公务卡制度等改革，大力推进财政预算决算公开。进一步强化审计监督，维护良好的财经秩序。

(二)加快调整优化经济结构，在构建现代产业体系上迈出新步伐。

加快发展现代农业，促进农业结构优化升级。抓住我市被列为国家现代农业示范区的机遇，围绕农业增效、农民增收，着力提升农业现代化、科技化水平。大力开展粮棉菜高产创建活动，提高主要农产品供给保障能力。围绕渔业、畜牧业等特色优势产业，大力发展园区农业，重点搞好现代渔业示范区、高效生态农业示范区和现代畜牧业示范区建设，扶持发展10个现代农业示范园区。深入推进农业产业化经营，重点实施泰国正大集团现代农业科技生态园、大地乳业万头奶牛养殖、澳亚现代牧场等30个过亿元项目；扶持30家重点农业龙头企业加快发展，新发展农民专业合作社100家、培育示范社50家。继续抓好黄河口优势农产品品牌培育，加强农产品质量安全监管。加大农业科技创新与推广力度，抓好中德农牧业技术培训中心建设，推动高效生态农业示范区创建国家农业科技园区；完善基层农技推广体系，多形式开展农业科技培训，培育新型农民。认真落实各项强农惠农富农政策，大力发展农村二、三产业，持续增加农民收入。加强农业农村基础设施建设，加快黄河引水、灌区配套与节水改造、南水北调等水利工程建设，新改建引水泵站2座，疏浚治理河道74公里，田间节水改造4.6万亩，改造中低产田1.2万亩，进一步提升农业机械化、信息化水平，提高农业综合生产能力；新建改造农村公路320公里，继续实施农村饮水安全、清洁能源工程，搞好农村电网续建配套，改善农村人居环境。

加快发展高新技术产业和战略性新兴产业，促进工业结构优化升级。改造提升现有优势产业，以石化、橡胶轮胎、造纸、纺织、有色金属产业为重点，用高新技术嫁接改造，积极推进整合重组，促进产业向高端发展。石化产业，加强与中石化、中海油的战略合作，重点抓好胜利石化总厂扩建、石大科技集团搬迁改造、中海油500万吨石化等大项目，推动产业向园区集中，逐步形成完整的产业链。橡胶轮胎产业，着力抓好技术研发、品牌培育和流通体系建设，扶持骨干企业做大做强，提高市场竞争力和可持续发展能力。着力突破先进制造业，以汽车及零部件、石油装备产业为重点，加快推进广汽吉奥汽车、德国KH发动机、高端石油装备产业园等大项目，拉长产业链条，壮大产业集群。积极培育战略性新兴产业，以电子信息、新能源和新能源汽车产业为重点，加快推进飞利浦3D电视、新加坡天安基集团平板电脑、中美新能源合作示范产业园、山东胜通光学薄膜、科岭增程式电动车等大项目，尽快形成规模优势。深入推进“两化”融合，突出抓好20个重点企业和重点项目建设，支持企业运用信息技术提升自动化、智能化、管理现代化水平。

加快发展现代服务业，促进三次产业结构优化升级。以大项目为支撑，加快构筑高端服务业产业链，打造黄河三角洲服务中心和服务业聚集区。加强服务业重点城镇、园区、企业和项目建设，重点抓好黄河口文化产业基地、黄河三角洲动物乐园等大项目，尽快壮大服务业规模，提高发展水平。优先发展文化旅游业，加快推进重点景区开发。黄河口生态旅游区重点实施游客服务中心、鸟类救护科普中心和木屋休闲区三大工程，天鹅湖旅游度假区重点实施筑岛、坝体绿化改造工程，孙子文化旅游区重点实施主题公园工程；规划建设黄河三角洲旅游集散中心，加大宣传推介力度，打造知名旅游品牌。着力突破现代物流业，重点抓好中海油物流园区、万通海欣保税罐区、宝港国际海关监管罐区、黄河三角洲农资物流园区等大项目，培育一批大型物流企业；完善规划，统一布局各类市场。加快发展服务外包业，实施软件园二期工程，着力引进一批知名服务外包企业，尽快实现大的突破。积极发展金融保险业，打造区域性金融中心。落实房地产调控政策，促进房地产业健康发展。改造提升商贸餐饮等生活性服务业，加快发展商务服务、会展、休闲健身等新兴服务业。

(三)着力推进城市和基础设施建设，在打造秀美宜居城市上展现新形象。中心城在加快推进大剧院、城市规划展览馆、科技馆、奥体中心等续建工程的同时，重点抓好四大工程：一是城市道路及沿街立面改造工程。东城重点抓好东三路、胶州路等4条道路改造，对黄河路、胜利大街等5条道路进行沿街立面改造，启动建设东八路南延工程，按照集会广场标准对新世纪广场进行综合改造；西城重点抓好西四路、济南路及沿街立面改造。二是城市旧区改造工程。东城重点抓好“三大片区”提升改造；西城重点实施油田会议中心、中心医院、胜动集团等区块改造。三是城市公园建设工程。新建改造明月湖公园、秋月湖公园等10处城市公园，为市民提供更多休闲空间。四是市政公用设施工程，实施南郊水厂扩容等一批供水、供热设施项目。理顺中心城管理体制，建立完善数字化城管系统，提高城市管理科学化、精细化水平。加强县城和小城镇规划建设管理，加大城中村、城郊村、滩区村改造力度。

加强重大基础设施建设。加快突破东营港，年内完成中海油2&times;5万吨级、2&times;5千吨级液体化工码头、宝港国际2&times;5千吨级精细化工码头、万通2&times;2万吨级液体化工码头和公共管廊带建设，中海油自用管廊达到投用条件；实施内港池综合整治，启用南港池。进一步完善提升广利港规划，年内开工建设。配套完善胜利机场基础设施，增开航线航班。加快新博公路和德大铁路建设进度，推动东济高速公路、沿海高等级公路、黄大铁路、寿平铁路尽快开工。加快推进东营港防潮堤建设，启动建设滨海生态城防潮堤。加强能源建设，推动大唐东营电厂项目取得实质性进展，建设一批风力发电和光伏发电项目，完成东营至潍坊天然气管线工程。

(四)加强节能减排和环境保护，在生态环境建设上增创新优势。

全面加强生态建设。突出抓好总投资25亿元的龙居、丁庄等10处生态林场建设，完成造林面积8万亩；继续实施“三网”绿化工程，抓好重点水系、干线道路、示范片区绿化和沿海防护林建设；大力提高城市绿化水平，高标准建设城市森林公园。实施金湖银河生态工程，年内完成主体河道和景观桥工程，引黄河水入城，打造城市生态水网水系。加强湿地生态保护，对东八路湿地进行改造绿化、完善配套设施，搞好自然保护区15万亩湿地修复和淡水补给工程。加强海岸带综合管理，抓好陆源污染、海上作业污染防控，提升近海环境质量。深入开展生态文明乡村建设工作，推进城乡环卫一体化，搞好村容村貌综合整治，改善农村生态环境。

深入推进节能降耗。以企业节能为重点，加强对50户重点耗能企业的监管，严格落实能评、环评和监测预警制度，积极推广先进节能技术产品和设备，实施好16个节能项目。加快淘汰落后产能。积极推行合同能源管理，以化工、造纸、纺织、橡胶轮胎等产业为重点，提高企业节能技术装备水平。全面落实最严格的水资源管理制度，建设节水型社会。大力发展循环经济，推行清洁生产，完善石油化工、造纸等10个循环经济产业链条，培育一批循环经济园区和生态工业示范园区。加快推进建筑、交通领域节能。完善公共机构能耗监测体系，建立绿色采购制度，推进节约型机关建设。积极创建省级低碳生态示范城市，重点抓好30个示范项目建设。

强力实施污染治理。全面实施公共环境改善工程，抓好小清河等省控重点河流、化工企业集中区和城区燃煤锅炉综合整治，开展大气污染物监测预警，强化废气点源面源管理，严控城市扬尘、机动车尾气污染，将PM2.5纳入常规空气质量监测体系。加大重点污染企业监管治理力度，开展新一轮重点流域排污企业限期治理工作，依法取缔土小污染企业。严格环境执法，严肃查处偷排偷放、超标排放、恶意排污等违法行为。加强环境基础设施建设，加快推进西城南、西城北等10座污水处理厂、生活垃圾焚烧发电、城市污泥综合处置等项目建设，实施好7座污水处理厂提标和西城雨污分流改造工程。基本完成中心城工业企业搬迁改造。着力解决重金属、农村面源污染等损害群众健康的突出环境问题。

(五)深化重点领域改革，在体制机制上激发新活力。着力推进金融创新，加快组建黄河三角洲产权交易中心，培育壮大汽车、石油装备等产业投资基金，引进大型融资租赁公司，合作成立大型担保公司，稳步发展小额贷款公司，积极培育面向小微企业和“三农”的金融机构。深化投资体制改革，规范政府投融资平台运行，全面提升运作能力。促进民间借贷规范化发展，依法打击非法集资，防范金融风险。扎实开展未利用地开发管理改革试点，抓好东营黄河三角洲综合开发有限公司运营管理，加快推进总规模30万亩的8个未利用地开发项目建设；探索建立耕地占补平衡指标市场交易机制，稳步推进城乡建设用地增减挂钩试点；搞好矿区土地联合开发政策研究，统筹安排城市建设和油气开发用地。深化农村综合改革，规范和促进土地承包经营权流转，年内完成农村集体土地所有权确权登记颁证；继续深化集体林权制度配套改革。加快供销社改革发展。扩大县区和开发区事权，搞好大王等经济发达镇行政管理体制改革试点。稳步推进事业单位分类改革。积极争取资源型城市可持续发展试点，加快建立生态补偿机制，探索资源有偿使用制度。

(六)实施科教兴市和人才强市战略，在创新型城市建设上达到新水平。

优先发展教育。以率先实现教育现代化为目标，深化教育改革，加大教育投入，促进各级各类教育均衡发展。加快推进城乡中小学办学条件标准化、校舍安全工程和幼儿园标准化建设，改善办学条件。调整优化中心城区教育布局，迁建市特殊教育学校、市中等专业学校，改造提升市实验学校，抓好金融小学、锦苑小学续建和胜利五中扩建。实施教育惠民工程，对全市在园家庭经济困难幼儿、孤儿、残疾幼儿给予保育费补助，免除城镇义务教育阶段学生的课本费、作业本费，为全市低保家庭学生免费提供学生装，对农村义务教育阶段学生给予营养补助。加强校车安全和校园环境治理。高度重视职业教育，支持东营职业学院建设国家骨干高职院校，加快市技师学院二期工程建设。支持中国石油大学东营校区办成高水平大学。积极引进高等教育资源，吸引国内知名高校来我市办学或设立教学实践基地，深入推进与山东大学战略合作。鼓励和规范民办教育发展。加强教师队伍建设，推进素质教育，提高教育教学质量。

推进科技创新。积极构筑创新平台，加快建设黄河三角洲国家可持续发展研究中心、山东大学东营研究院，依托国家大学科技园创建国家级高新区，引进一批高层次研发机构，加强应用基础研究和公共技术攻关。围绕产业发展重点领域，强化协同创新，优化资源配置，集中实施一批重大科技专项，新建一批产业技术创新联盟，提升产业核心竞争力。发挥企业创新的主体地位，引导企业加大研发投入，支持企业建设工程技术研究中心、企业技术中心，重点抓好国家采油装备工程技术研究中心建设。加强产学研结合，提高科技成果转化率和产业化水平。

加强人才队伍建设。完善人才政策，持续增加人才投入，加大人才培养引进使用力度。拓宽人才培养渠道，以高层次人才和高技能人才为重点，统筹抓好各类人才队伍建设。创新引才用才机制，深化“人才特区”建设，打造黄蓝经济区人才高地。着力抓好“黄河三角洲学者”、“金蓝领”培训等重大人才工程，引进一批高层次海外人才、创新型科技人才和产业领军人才，带动高端产业发展。健全人才公共服务体系，强化创业人才政策扶持，加大知识产权保护和开发力度，营造人才创新创业的良好环境。

(七)繁荣发展先进文化，在文化强市建设上实现新突破。坚持社会主义先进文化前进方向，以高度的文化自觉和文化自信，大力推进文化改革发展，加快建设文化强市，提升东营文化的竞争力和影响力。加强社会主义核心价值体系建设，不断巩固全市人民团结奋斗的思想基础。扎实推进全国文明城市创建工作，广泛开展群众性精神文明创建活动，提高全社会文明程度。加快发展文化事业，坚持政府主导，加大投入，构建覆盖城乡、功能完善的公共文化服务体系。深入实施文化惠民工程，加快推进图书馆、群众文化艺术馆、黄河文化博物馆、乡镇综合文化站、村文化室等公共文化设施建设。繁荣发展文艺创作，推出更多具有黄河口地域和时代特色的文艺精品。做好第十届中国艺术节筹备工作。大力发展文化产业，促进文化与经济、旅游、科技深度融合，积极发展文化创意、动漫游戏等新兴文化产业。加快培育一批骨干文化企业，支持市黄河文化传媒集团加快发展。深入挖掘孙子文化、吕剧文化、石油文化、生态文化内涵，开发文化价值，推进传承创新，着力打造黄河口特色文化品牌。积极发展健康向上的网络文化。深化文化体制改革，激发文化发展活力。加强文化市场综合执法，整顿文化市场秩序。繁荣发展哲学社会科学。加强民主法制建设，扎实推进“六五”普法和依法治市。

(八)着力保障改善民生，在社会建设上开创新局面。

努力推动就业创业。坚持更加积极的就业政策，统筹抓好重点群体就业，确保全年新增城镇就业3.1万人，农村劳动力转移就业3.6万人，保持就业形势基本稳定。重视发展劳动密集型产业，多渠道开发就业岗位，千方百计扩大就业容量。把促进高校毕业生就业放在首位，加快建设大学生就业创业服务中心，落实各项扶持政策，引导毕业生到基层就业和自主创业。全面实施创业引领计划，落实创业富民政策，以创业带动就业。加快整合人才市场和劳动力市场，加强基层公共服务平台建设，不断完善公共就业服务体系。加强劳动争议调解仲裁和劳动监察，积极构建和谐劳动关系。完善社会保障体系。全面推进城乡居民养老保险，城乡居民基础养老金由每月60元提高到120元，比国家规定的标准高1倍多；完善企业养老保险接续办法。不断提高医疗保障水平，城镇居民医保、新农合财政补助标准提高到260元，增加城乡居民大额医药费财政补助，完善医疗保险异地就医办法。健全被征地农民、城镇未参保集体企业退休人员等特定群体社会保障制度，适时提高城乡低保、农村五保供养标准，加大城乡弱势群体、困难群众和优抚对象的基本生活保障力度。积极发展社会福利、慈善和养老事业。实施城乡安居工程，年内建设各类保障性住房1万套，改造棚户区住房1500户；支持胜利油田、石油大学、济军基地职工住房建设；适时启动农村安居工程，改造农村危房，建设农村特困群众安居房。

大力发展社会事业。继续深化医药卫生体制改革，扩大公立医院改革试点范围。支持市人民医院创建“三甲”医院，规划建设急诊急救中心。抓好市疾病预防控制中心建设。健全完善基本药物制度，规范基本药物配备使用。推进基层医疗卫生机构综合改革，规范乡村医生队伍管理。积极扩大免费医疗试点。深入开展全民健身运动，加快竞技体育发展，办好黄河口国际马拉松赛等大型赛事。做好人口计生工作，稳定低生育水平，努力提高人口素质。扎实开展双拥共建，做好国防教育、人民防空、民兵预备役等工作。关心支持妇女儿童、青少年、残疾人和老龄事业发展。进一步提高广播电视、新闻出版、外事侨务、民族宗教、无线电管理工作水平。积极推进档案、仲裁、气象、对台、史志、红十字会、防震和救灾减灾等工作。继续做好对口支援工作。

维护社会安全稳定。组织开展“社会管理创新突破年”活动，加强社区建设，完善基层社会管理服务体系，建立健全共同治理新机制，推进社会管理法治化、规范化。更加注重从源头上预防和减少社会矛盾，切实解决群众合法合理诉求，坚决纠正损害群众利益的行为。加强和改进信访工作，排查梳理信访积案，着力解决影响稳定的突出问题。完善社会治安和油区治安防控体系，深入开展严打整治斗争和打霸治痞行动，切实增强人民群众的安全感。进一步加强安全生产管理，严格落实责任制，全面排查和消除安全隐患，加大重点行业和领域整治力度，有效防范和坚决遏制重特大安全事故发生。强化食品药品安全监管，确保人民群众饮食用药安全。加强公共安全突发事件应急管理，提高预防预警和处置能力。

各位代表，民生大于天，为老百姓谋福祉是政府义不容辞的责任。我们一定尽最大努力，投入更多财力，着力解决人民群众最关心、最直接、最现实的利益问题，让全市人民生活得更加幸福美好！

四、切实加强政府自身建设

面对形势发展的新要求和全市人民的新期盼，全体政府工作人员要以更加昂扬的斗志、更加务实的作风、更加有力的举措，高标准做好各项工作，全面提升政府执行力和公信力，肩负起历史赋予的重任。

要坚持为民执政。牢固树立群众观点和群众立场，始终保持与人民群众的血肉联系，倾听群众呼声，体察群众疾苦，反映群众愿望，真心实意为群众谋利益。牢固树立正确的政绩观，把人民幸福作为政府工作的第一目标，把群众满意作为政府工作的第一标准，多做顺民意、解民忧、惠民生的实事好事。畅通社情民意渠道，抓好市长公开电话、行风热线、政务微博建设，及时解决各种利益诉求，主动接受群众监督。实施好十大民生工程，严格目标责任，狠抓推进落实，确保按期完成。

要坚持务实从政。把解放思想作为推进工作的源动力，不断提升思想境界、提高工作标准，始终保持奋发有为的精神状态，敢闯敢试，敢为人先，努力开创各项工作的新局面。大力弘扬求真务实作风，坚持一切从实际出发，察实情、办实事、求实效。落实为民服务举措，主动深入基层、深入群众，加强对群众最盼、最急、最忧问题的调查研究，找准要害、解决问题。加强行政效能建设，规范办事程序，简化办事环节，压缩会议文件，推动工作提速增效。加大抓落实的力度，完善目标管理责任制，加强督查考核，对重点工作、重点项目，细化分工，明确责任，一抓到底，落到实处。牢固树立全市一盘棋的思想，自觉维护大局，加强协调配合，提高整体战斗力。

要坚持依法行政。扎实开展“行政程序年”活动，深入贯彻依法行政实施纲要，全面落实山东省行政程序规定，规范行政行为，建设法治政府。积极推进依法科学民主决策，规范行政决策程序，实行重大决策听证和合法性审查制度，完善行政决策风险评估机制，加强重大决策跟踪反馈和责任追究。严格行政执法责任制，严肃查处违法乱纪行为。深化行政审批制度改革，清理和规范行政审批事项，完善“两集中、两到位”运行机制。深入推进政务公开，所有面向社会服务的政府部门都要全面推行办事公开制度。进一步加强电子政务建设，抓好政府门户网站、便民服务网络平台建设，推行网上审批，更加方便群众办事。自觉接受人大法律监督和政协民主监督，高度重视新闻舆论和社会公众监督，广泛听取社会各界的意见和建议。

要坚持廉洁勤政。认真落实党风廉政建设责任制和廉政准则，健全完善惩治和预防腐败体系。深入开展反腐倡廉教育，筑牢拒腐防变的思想道德防线。完善反腐倡廉制度体系，坚持用制度管权、管事、管人，加强对权力运行的监督和制约。深入推进专项治理工作，继续深化工程建设领域突出问题专项治理，深入开展庆典、研讨会、论坛过多过滥问题和公务用车问题专项治理。加大违纪违法案件查办力度，依法严惩腐败分子。大力发扬艰苦奋斗的优良传统，坚持勤俭办事业，严格控制“三公”经费，切实降低行政成本。全体政府工作人员要加强学习，全面提高素质能力，带头创先争优，努力成为政治坚定、业务精通、清正廉洁、甘于奉献的人民公务员。

各位代表，新的征程已经开启，新的使命催人奋进。让我们更加紧密地团结在以胡锦涛同志为总书记的党中央周围，高举中国特色社会主义伟大旗帜，以邓小平理论和“三个代表”重要思想为指导，深入贯彻落实科学发展观，在省委、省政府和市委的坚强领导下，坚定信心，振奋精神，锐意进取，扎实工作，为推动全市经济社会又好又快发展、开创生态文明典范城市建设新局面而努力奋斗！
